# Implementation barriers in Singapore’s community-based falls prevention programmes: a root cause analysis

**DOI:** 10.3389/fpubh.2026.1913505

**Published:** 2026-10-12

**Authors:** Catherine Wen Huey Lim, Vanessa Koh, Jing Wen Goh, Lakkhina Troeung, Atiqah Lee, Malcolm Ravindran, David B. Matchar, Reshma Merchant, Angelique W. M. Chan

**Affiliations:** 1 Centre for Ageing Research & Education, Duke-NUS Medical School, National University of Singapore, Singapore, Singapore; 2 Programme in Health Services Research & Population Health (HSRPH), Duke-NUS Medical School, National University of Singapore, Singapore, Singapore; 3 Department of Medicine (General Internal Medicine), Duke University, Durham, NC, United States; 4 Division of Geriatric Medicine, Department of Medicine, National University Hospital, Singapore, Singapore

**Keywords:** current reality tree, falls prevention intervention, implementation barriers, older adults, root cause analysis

## Abstract

**Introduction:**

Falls are a critical public health challenge for ageing populations. Despite robust evidence supporting the clinical effectiveness of multi-component falls prevention programmes (FPPs), a pronounced implementation gap exists in community settings. This study investigates systemic barriers to delivering FPPs in Singapore, focusing on resource constraints, operational capacities, and scaling risks.

**Methods:**

This qualitative study employed a structured Facilitated Process Improvement workshop grounded in the Theory of Constraints. Fourteen management representatives from seven diverse Integrated Community Care Providers participated in the half-day workshop. The workshop featured three core activities: (i) capacity mapping of current and projected FPPs delivery capabilities, identification of (ii) challenges in falls risk screening processes and (iii) barriers in programme monitoring and evaluation. Workshop discussions were moderated through probing and clarifying using Categories of Legitimate Reservation, a logical framework to ensure all identified challenges were clearly defined and the causal linkages proposed were logically sound. Workshop insights were systematically analysed through the construction of a Current Reality Tree to map fundamental constraints.

**Results:**

The core undesirable effect was the continued prevalence of avoidable injurious falls. Root causes identified included: (1) no systematic effort to inform older adults about falls and falls prevention, (2) community provider lack of training resources, (3) community provider insufficient funding, (4) a lack of both formal and informal data on demand for FPPs, and (5) high manpower cost for vendors to conduct FPPs. Findings from the CRT analysis are organised into three themes: (a) older adult perception and awareness, (b) provider operational capacities, and (c) inconsistent vendor supply. Findings reveal three systemic gaps: an information gap obscuring need, a resource/capacity gap crippling delivery, and an economic gap making programmes unsustainable.

**Implications:**

Effective scaling requires moving beyond funding discrete programmes to investing in the foundational pillars of the preventive health ecosystem in Singapore. Addressing information gaps, resource, and economic gaps identified in this study is a necessary step towards making falls prevention a scalable and sustained reality.

## 1 Introduction

Falls among older adults are a major public health burden in ageing populations worldwide, with approximately one in four community-dwelling older adults aged 65 and above experiencing a fall annually (1). The risk of falls increases with age, with one in two older adults aged 80 and above falling each year (2). The consequences of falls extend beyond physical trauma to include serious injury, hospitalisation, long-term functional decline, fear of falling, loss of confidence, and increased dependency and social isolation (3).

Multi-component falls prevention programmes (FPPs), which incorporate one or more evidence-based components such as strength and balance training, medication review, and home hazard modification, have been shown to be effective in reducing falls incidence among older adults (4, 5). In contrast to the group-based exercise programmes commonly found in community settings, FPPs are tailored for the heterogeneous high-risk population by addressing the risk factors, such as muscle weakness, balance impairments, environmental hazards, cognitive decline and polypharmacy, falls education that contribute to falls (6, 7). Reviews show that FPPs lower the rate of falls by 28 to 38% annually and reduce both physical and psychosocial fall-related burden (8–13). However, the real-word impact of FPPs extends beyond clinical effectiveness and depends largely on successful community implementation, long-term sustainability, and continued participant engagement.

The successful translation of evidence-based interventions into routine practice is also highly context dependent (14). Existing research demonstrates the feasibility of implementing FPPs in community settings such as local community centres, where accessibility and engagement can be enhanced (13). For example, research on nationwide older adult wellness programmes in Japan and United States, has shown that leveraging a network of government-supported community centres enables the rollout of a standardised, evidence-based curriculum, which in turn promotes higher and more consistent participation rates among the ageing population (15, 16). However, variations in funding mechanisms, workforce composition, and organisational priorities mean that international models cannot be adopted directly in other countries (17–19). This has contributed to a pronounced translation gap between robust evidence and sustained community practice.

Singapore, a rapidly ageing nation, exemplifies this global challenge. Singapore faces a significant and growing burden from falls, where 85% of geriatric emergency department attendances are fall-related (20). Several FPPs and related healthy ageing programmes are available in Singapore, though these are run by region-specific organisations and are mostly targeting improvement in overall physical health (see Supplementary Table S1 for summary). Active Ageing Centres (AACs) are the primary community touchpoints for community-dwelling older adults in Singapore, with over 220 centres nationwide reaching 80% of the older adult population (21, 22). AACs are designed to provide information and programs that promote healthy ageing. Thus, AACs present a prime platform for delivering FPPs due to their established reach and infrastructure. However, while AACs widely offer group-based exercise programmes, these often lack the targeted components proven most effective for falls prevention (5, 12).

This discrepancy identifies a critical research gap for investigating the practical challenges of implementing FPPs from the perspective of community providers, including specific organisational needs, operational constraints, and perceived risks. Using a root cause analysis framework, this study aims to uncover the underlying systemic issues inhibiting the delivery of FPPs across AACs in Singapore. The findings will provide crucial insights for scaling FPPs locally and contribute to the broader understanding of implementing complex interventions in community-based settings globally.

## 2 Methods

### 2.1 Study design

A Facilitated Process Improvement (FPI) workshop was conducted with management representatives of community providers in January 2025. The aim of this workshop was to identify systemic barriers to implementing FPPs that apply across AAC contexts, though possibly of different types or degrees in a given AAC. This workshop represented the first step in a broader FPI process aimed at tailoring solutions to contextual variation, with the current work focused on identifying the functional activities necessary for success, thereby laying the foundation for subsequent site clustering and the development of a context-sensitive toolkit. The FPI approach provided a structured setting where facilitators guided the stakeholders in the construction of a Current Reality Tree (CRT) to a causal logic map describing the interrelated causes of a fundamentally undesirable effect (23)—here the prevalence of avoidable falls. This root cause analysis framework moves beyond descriptive barriers to identify the underlying drivers of the crucial system features leading to implementation failure.

### 2.2 Participants

Management staff from Integrated Community Care Providers (ICCP), who coordinate the AACs, were invited to participate in a half-day workshop. Out of the 56 ICCPs in Singapore, seven were purposefully selected due to their previous initiatives and interests for falls and frailty prevention. Of the seven, three were large ICCPs that had of total of over 60 AACs all over the nation. A total of 14 stakeholders from seven ICCPs, all with experience in running programmes similar to FPPs—such as group-based exercise programmes—participated in the workshop to ensure diversity in reach (number of residents served), resources, and focus areas (activities provided). The stakeholders consisted of clinicians (a geriatrician and a physiotherapist), senior leaders (chief executive officers and directors), and community managers. These stakeholders had extensive experience in falls and/or frailty prevention programmes within their respective ICCPs and helmed the roles of designers, implementers and funders of such programmes. All stakeholders took part in the entirety of the workshop.

### 2.3 FPI workshop activities

The FPI workshop was structured into three core activities as illustrated in Table 1: (i) capacity mapping (Activity 1), (ii) identification of challenges in falls risk screening (Activity 2), and (iii) identification of challenges in monitoring and evaluation (Activity 3). The FPI workshop was structured into three core activities as illustrated in Table 1: (i) capacity mapping (Activity 1), (ii) identification of challenges in falls risk screening (Activity 2), and (iii) identification of challenges in monitoring and evaluation (Activity 3). To promote an ongoing productive critique of the CRT, the stakeholders apply a guide – the Categories of Legitimate Reservation (CLR).

**Table 1:** Structure and objectives of the FPI workshop activities.

Activity	Description	Objective
1. Capacity mapping	A group activity where the stakeholders, guided by a structured worksheet (Supplementary Figure S1), described their current operational capacity and projected scaling potential for FPPs over a 1–2 year timeline. This was followed by a round-robin sharing session.	To benchmark the current state of AAC capacities and contextualise subsequent discussions on barriers based on their respective organisational realities.
2. Challenges in falls risk screening	A moderated roundtable discussion focused on identifying barriers to screening older adults for falls risk. The stakeholders generated ideas individually on sticky notes, followed by a group discussion to explore each challenge and its relationships.	To identify and understand the specific constraints and root causes within the falls risk screening pipeline.
3. Challenges in monitoring and evaluation	A moderated roundtable discussion focused on barriers to evaluating FPPs success. Stakeholders used colour-coded sticky notes to differentiate between challenges in monitoring and evaluation and potential indicators of success, followed by a root cause analysis.	To identify barriers to effective programme evaluation and to explore relevant indicators for measuring FPPs success from a provider perspective.

### 2.4 Categories of legitimate reservation

Workshop discussions were moderated using CLR, a logical framework from the Theory of Constraints used to validate cause-and-effect relationships in system models (24, 25). The CLR contains 8 different reservations—clarity, entity existence, causality existence, cause-effect reversal, indirect effects, insufficient cause, additional cause, and tautology—each addressing a specific way in which a causal claim may be flawed (26). Facilitators applied this framework to ensure all identified challenges to implementation were clearly defined and that the causal linkages proposed between them were logically sound and evidence-based, rather than correlative or assumed. This structured process of probing and clarification continued for each activity challenge until a coherent causal narrative was articulated and agreed upon by the group, thereby ensuring the robustness of the qualitative data generated. A summary of the eight CLR categories is provided in Supplementary Table S2. Illustrative examples of causal linkages validated through the CLR process during the workshop, including the reservation type applied and the resulting revision, are presented in Supplementary Table S3.

#### 2.4.1 Activity 1: capacity mapping

In this activity, the stakeholders worked in their organisations to complete a structured activity worksheet (Supplementary Figure S1), responding to the prompt that asked them to estimate the number of participants their organisation could accommodate given current resources (e.g., staffing, space, equipment): “At what scale can your organisation implement falls prevention programmes?” To ground their estimates in existing operations, they were instructed to base their estimates on direct FPP experience (either independently or via research partnerships), and to use their current delivery of group-based exercise programmes as a practical reference point if such experience was lacking. A facilitator was assigned to each table to guide the discussions using the CLR to help the stakeholders clearly define capacity factors and scrutinise the causal linkages in their scaling plans (24). Following this, each organisation was invited, in a round-robin fashion, to share their profile of current capacity and near-term (1–2 year) scaling potential for FPPs, providing a foundational context for the subsequent activities.

#### 2.4.2 Activity 2: identification of challenges in screening

The session began with the prompt: “To implement FPPs at scale, what are the challenges you face when screening for falls risk?”. The stakeholders were first given 5 mins to individually generate and record each challenge on a separate sticky note. This was followed by a structured, round-robin discussion in which each of them selected a challenge to elaborate on. As in the previous activity, facilitators used CLR to guide the discussion, probing responses to clarify the nature of each challenge and to map the cause-and-effect relationships between them. A challenge was considered sufficiently clarified when a coherent narrative and hypothesis regarding its cause and effect on implementation could be articulated and agreed upon by the group. This process was repeated for all challenges to identify areas of agreement, uncover underlying assumptions, and build a consensus on their root causes before proceeding to the next activity.

#### 2.4.3 Activity 3: identification of challenges in monitoring and evaluation

The final structured activity began with a new prompt: “What are the challenges you face in monitoring and evaluating the success of falls prevention programmes?”. As before, the stakeholders used colour-coded sticky notes to differentiate between challenges and potential indicators of success. A subsequent round-robin discussion, once again guided by the CLR, followed to identify the root causes of the monitoring and evaluation constraints and to explore the utility and feasibility of the proposed success indicators.

The workshop proceedings were audio-recorded with stakeholders’ consent. Concurrently, members of the study team took detailed written notes to capture key discussion points. The audio recordings were later transcribed verbatim for subsequent analysis.

### 2.5 CRT construction

CRT is a cause-effect logic tool within the Theory of Constraints that depicts a verified model of a system (25). The logic of the tree is verified by systematically applying CLR to challenge and verify each cause-and-effect linkage, ensuring the final model is robust and non-contradictory. It is employed as a structured tool to conduct gap analysis by systematically mapping root causes of problems and identifying leverage points (27). A CRT consists of nodes, which at the top of the tree is the fundamentally Undesirable Effect (UDE)—the outcome that is not undesirable simply for what it may cause—intermediate effects (the factors that lead to the UDE), and the root causes (the underlying problem(s) or system constraints). The UDE is identified based on the main objective of this study. Meeting summary notes from the discussions were reviewed to identify statements describing problems, constraints, or negative outcomes; these were extracted as and served to populate a causal web, represented as the CRT.

The CRT was formed from the UDE (top) down to the root causes (bottom); each node was positioned within the tree and connected using cause-effect logic, working backwards from observed effects to underlying causes. The mapping was conducted iteratively to establish logical connections between each node: an ‘and’ connects multiple causes that are jointly necessary for the effect to occur—if any one cause was absent, the effect would not manifest; an ‘or’ connection shows the relationship where any single cause was sufficient on its own to produce the effect; and a ‘magnitude’ connects independent causes that, while not strictly necessary together, compound to amplify the severity or scale of the outcome. The structure was refined by merging duplicate entities and grouping similar UDEs to eliminate redundancies. The process continued until deeper causal layers were achieved and no further underlying causes were identified.

The resulting CRT was reviewed for internal consistency and verified against the original meeting notes to ensure that all key UDEs were represented and logically linked. Furthermore, to ensure each cause-effect connection was empirically supported, the relationships between nodes were validated against relevant literature where available. For relationships that could not be validated due to an absence of prior empirical discussion in the literature, these were retained based on stakeholder consensus, reflecting the experiential knowledge of participants with direct operational experience. Where tensions existed between stakeholders’ perspectives and the literature, stakeholders’ input was prioritised to capture ground-level implementation realities that may not yet be well-documented.

## 3 Results

The ultimate UDE identified in this study was the continued prevalence of avoidable injurious falls among older adults (Supplementary Figure S2). The CRT identified two main contributing factors: a lack of community programmes to reduce falls, and low uptake of existing FPPs. The CRT analysis traced the causal pathways to identify five fundamental root causes situated at the base of the tree: (1) no systematic effort to inform older adults about falls and falls prevention, (2) lack of training resources for AACs, (3) insufficient funding for AACs, (4) a lack of both formal and informal data on demand for FPPs, and (5) high manpower cost for vendors to conduct FPPs. Findings from the CRT analysis are organised into three themes, which are: older adult perception and awareness (blue), provider operational capacities (yellow), and inconsistent vendor supply (red).

### 3.1 Older adult perception and awareness

In the context of older adults, the stakeholders perceived that the continued occurrence of avoidable injurious falls was primarily driven by the low uptake of existing FPPs. Two UDEs were thought to directly contribute to this low uptake: older adults do not see the value of FPPs, or older adults are unaware of available FPPs (Figure 1).

**Figure 1:**
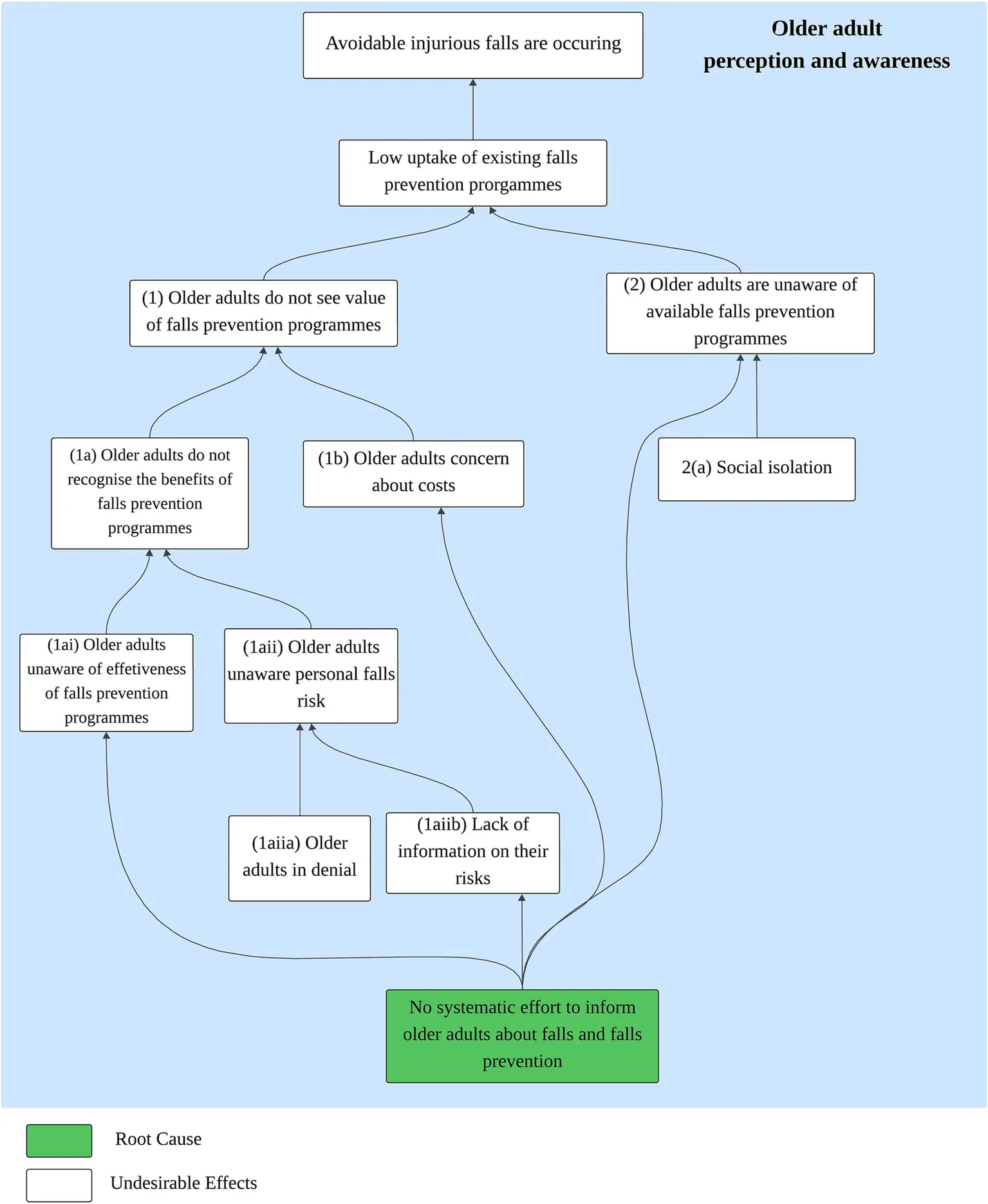
Sub-tree of CRT, illustrating older adults’ perception and awareness as barriers to falls prevention.

The perceived value of FPPs is low primarily because older adults do not recognise the benefits of attending FPPs, or they are concerned about costs of FPPs. Stakeholders identified that the prospect of paying for FPPs raises significant concern for older adults, as they must prioritise financial resources for daily needs and management of existing chronic health conditions. Furthermore, the failure to recognise the benefits of FPPs is due to older adults being unaware of the programmes’ effectiveness or of their personal falls risk. This lack of falls risk awareness is complicated by personal denial, which providers explained is a commonly observed psychological response to screening. The fear of being identified as high-risk for falls may convey a sense of hopelessness and fatalism, particularly among older adults who already believe falls are a normal part of ageing or simply a lack of information about their individual risk of falling.

Concurrently, the lack of awareness on programme availability is contributed by social isolation. These collective factors are ultimately driven by the root cause: the absence of a systematic effort to inform older adults about falls and falls prevention.

### 3.2 Provider operational capacity

In the context of provider operational capacity, stakeholders perceived that the occurrence of avoidable injurious falls persists primarily due to the lack of sustained FPPs in AACs (Figure 2). A lack of sustained programmes represents an absence of institutionalised, internally managed capability within the AACs to run them continuously, as noted by the stakeholders. Notably, while AACs regularly host group-based exercise programmes, these are typically delivered by external vendors and do not require adherence to a complex protocol, such as delivering evidence-based exercise regimens followed by longitudinal assessments of falls risk among older adults. This capability gap is directly caused by either a lack of skills within AAC or insufficient manpower. Alternatively, it may be caused by the combination of complex demands on AACs to meet funding requirements and FPPs not being included as a key performance indicator (KPI) by funders.

**Figure 2:**
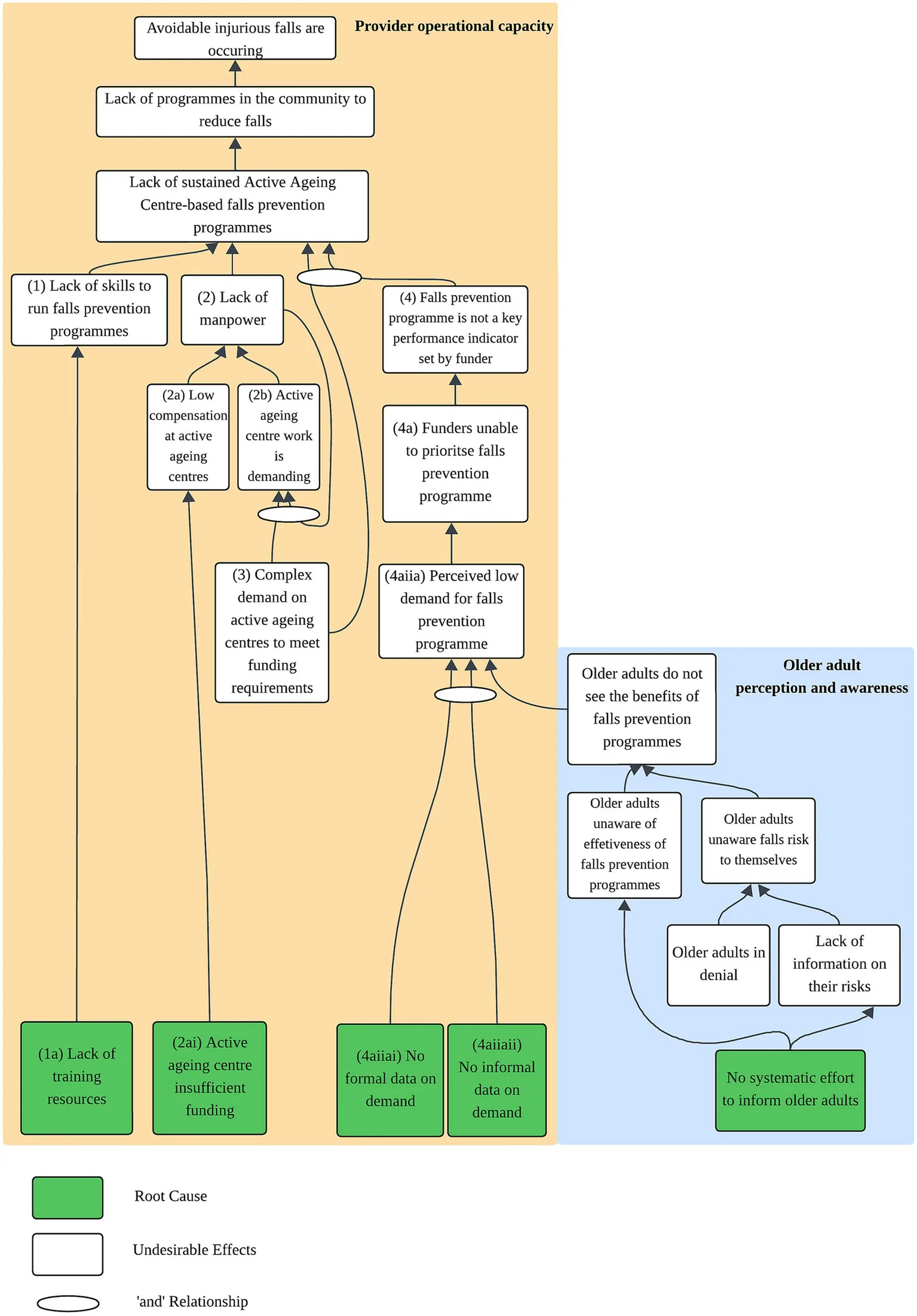
Sub-tree of CRT developed, illustrating provider’s operational capacity as barriers to falls prevention.

The root cause for the lack of skilled AACs staff to run FPPs is a lack of training resources which includes limited funding for training, a shortage of available courses, and inadequate support to cover routine work when staff attend training. As a result, staff are unable to independently deliver evidence-based FPPs, which require more specialised skills to safely cater to frail older adults at higher risk of falling. The lack of manpower within AACs is due to low compensation and the demanding workload of AAC. Specifically, low compensation stems from the root cause of insufficient funding for AACs. The demanding workload arises from a combination of factors, including the pre-existing lack of manpower and the overwhelming number of diverse demands that AACs must fulfil to meet funding requirements. As highlighted by the stakeholders, AAC staff must manage a wide array of tasks beyond standard older adult engagement, often assuming multiple roles, including addressing *ad hoc* issues such as resolving everyday household issues residents may face (i.e., plumbing or mechanical problems in residents’ homes).

The omission of FPPs as a KPI by funders is primarily attributable to funders unable to prioritise FPPs. ICCP stakeholders further elaborated that, within this context, competing KPIs, such as time-sensitive outreach targets and long-term preventive health goals, have largely consumed the proportion of time and resources needed to achieve primary performance metrics. Consequently, AACs face capacity constraints in implementing activities that fall outside these mandated metrics. Funders perceive a low demand for these programmes due to the absence of systematic data on FPPs demand, including both formal data (e.g., structured surveys, screening numbers) and informal data (e.g., staff observations, anecdotal feedback). This data gap critically impedes advocacy, as there is insufficient evidence to demonstrate programme need and secure funding. Compounding this perception is the complex issue relating directly to older adults being unable to recognise the benefits of FPPs, as discussed in the *Older Adults Perception and Awareness* section.

### 3.3 Inconsistent vendor supply

The lack of programmes in the community to reduce falls was also attributable to a shortage of vendors providing FPPs (Figure 3). ICCP stakeholders believed vendors are absent because FPPs are not seen as profitable. The perception of low profitability arises from a combination of factors: more profitable uses of vendor staff and perceived low per-person compensation, and perceived low demand for FPPs. The stakeholders noted that vendors, who often run multiple programmes concurrently, must prioritise how to allocate their limited manpower to maximise profits, making other programmes a more profitable use of staff time. Consequently, as the revenue generated per older adult enrolled in FPPs is low compared to other health or social programmes, vendors are incentivised to deploy staff to deliver more profitable projects, representing a more efficient use of their limited resources. Furthermore, the low demand for FPPs is directly linked to the variables of older adults being unable to recognise the benefits of FPPs and a lack of demand information, as discussed in the *Older Adult Perception and Awareness sections* and *Provider Operational Capacity*.

**Figure 3:**
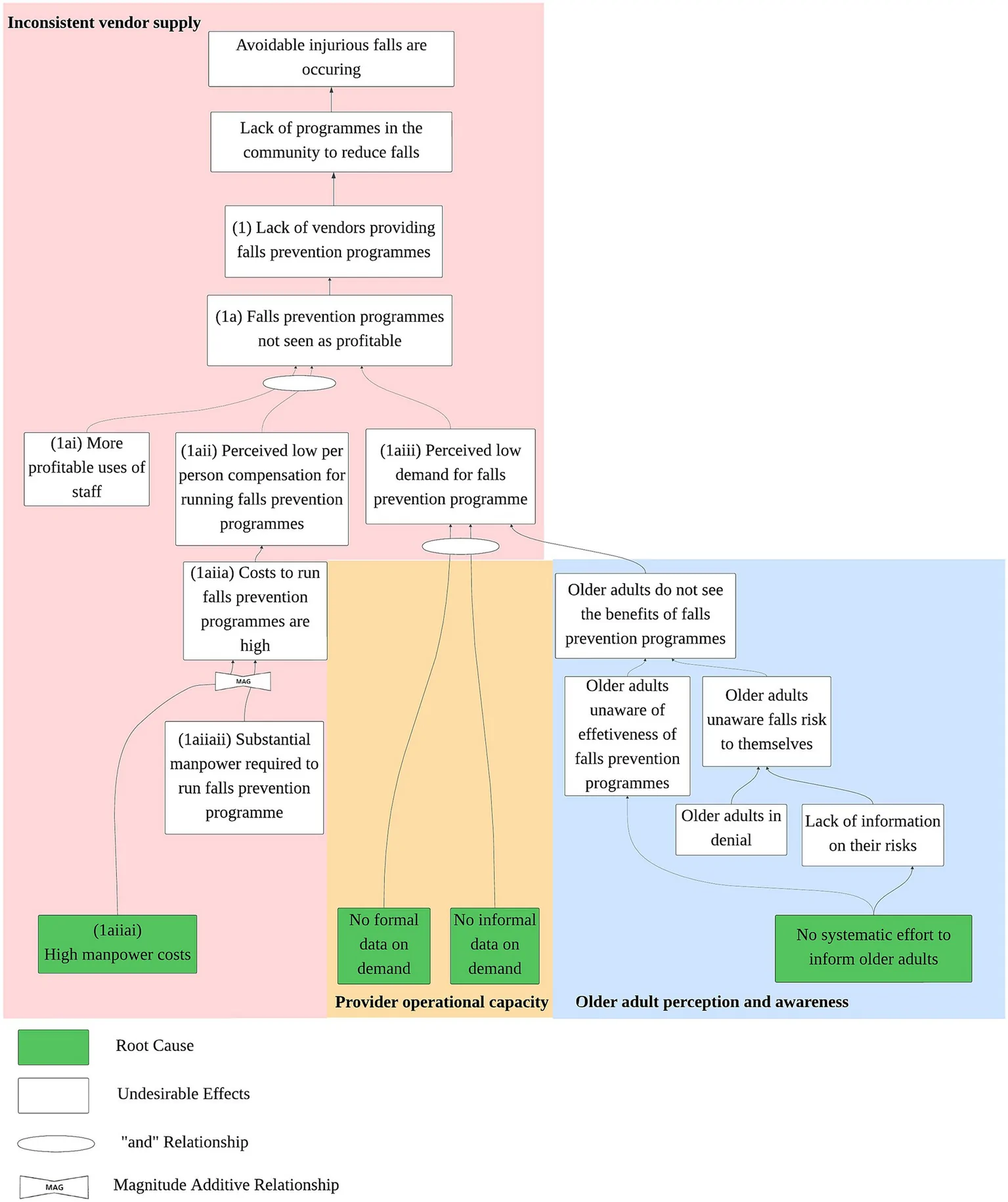
Sub-tree of CRT developed, illustrating inconsistent vendor supply as barriers to falls prevention.

The perception of low per-person compensation exists because the costs of running falls prevention programmes are high. These high costs are driven by high manpower costs and substantial manpower required. The stakeholders clarified that substantial manpower is necessary to run FPPs safely and effectively. This includes staff for arranging logistics as well as trained personnel to manage the high-risk participants during sessions. Furthermore, manpower costs are elevated as it is recommended that FPPs employ qualified clinicians or trained personnel to ensure safety and efficacy, including general practitioners to monitor health and conduct screenings on selected sessions, and physiotherapists to lead the exercise sessions and prevent injuries. While either factor alone increases costs, their compounding effect makes programmes financially unviable, as the substantial staffing needs and the high cost of each professional together impose an unsustainable financial burden.

## 4 Discussion

This study investigated the systemic constraints to scaling FPPs in Singapore by applying a root cause analysis framework to move beyond descriptive barriers and identify the underlying drivers of implementation failure. By mapping causal pathways across stakeholder groups, we delineated the fundamental root causes that prolong a cycle of low demand, limited supply and insufficient operational capacity. The five root causes collectively explain the observed challenges faced by providers, thereby providing a clear and actionable diagnosis of the systemic gaps that must be addressed to enable effective, scalable falls prevention.

A synthesis of the root causes reveals a foundational information and evidence gap, characterised by a lack of systematic outreach and a lack of demand data. This foundational gap directly fuels a resource and capacity gap among frontline providers, reflected in the providers’ lack of training and inadequate funding. These are compounded by an economic gap for vendors, driven by prohibitive manpower costs associated with delivering FPPs. Critically, these gaps are not linear but cyclical. For instance, the lack of demand data contributes to the lack of prioritisation of FPPs as a KPI, thereby suppressing programme supply and further limiting the generation of robust data on demand and effectiveness. This creates a vicious cycle where the perceived lack of programme value and demand for FPPs becomes a self-fulfilling prophecy.

These gaps are not unique to Singapore but represent a failure to establish universal enablers for scaling complex health interventions. Our findings align with constructs of the Consolidated Framework for Implementation Research, which emphasises that successful scaling of health interventions depends on supportive ecosystems (28). The information gap corresponds to deficits in the outer setting construct of *‘*Patient Needs & Resources*’*. The resource and capacity gap mirrors insufficient *‘Available Resources’* and a lack of ‘Access to Knowledge & Information’ within the inner setting of provider organisations. Finally, the economic gap highlights *‘Cost’* as a prohibitive intervention characteristic. The cyclical nature of the problem is explained by the interaction of these constructs across the inner setting (providers) and outer setting (funder policy and incentives) (29). The critical role of “evidence of value” and “demand data” in our findings emphasised the heightened need for economic and outcome-based justification in a resource-constrained environment.

The structural gaps identified in this study are not isolated but mirror those found in other rapidly ageing Asian societies striving to scale community-based preventive care. Evidence from China and Thailand reveals strikingly similar systemic constraints. In China, despite falls prevention being included in the National Essential Public Health Service Package since 2009, implementation is crippled by a foundational information gap: health administrators highlight a lack of local burden data as a reason falls prevention is not prioritised in workforce and budget allocation (30). Consequently, there is no dedicated budget, no relevant performance indicators, and no national action plan or guideline with specific evidence-based content for primary care, leading to a disconnect between policy rhetoric and implementable, funded programmes. Similarly, in southern Thailand, while falls are recognised as common and serious, community stakeholders report that falls are not prioritised as a public health concern (31). They explicitly call for “evidence-based strategies” and clearer, aligned government policies to justify stronger investment and coordinated action. These correspond with our findings on the resource and capacity gap, where although falls are recognised as a concern, there is a lack of dedicated resources for prevention activities. It also mirrors the cycle where a lack of policy priority curtails resource allocation, perpetuating the data and capacity gaps that pose ongoing barriers to progress.

The persistent lack of demand data to justify investment, combined with a funding structure incompatible with the safety-critical nature of falls prevention, creates a fundamental barrier to scale. Examining experiences from various countries provides critical counterpoint, illustrating how different systemic configurations can address the same fundamental challenges. These examples demonstrate that the barriers observed in Singapore are not inevitable but are amenable to strategic system-level intervention. For instance, directly tackling the foundational information gap, a study in Hong Kong ran a territory-wide electronic-health-record (EHR) based surveillance by assembling EHR of older adults to develop a 1-year falls-hospitalisation prediction model using routinely collection variables (e.g., demographics, diagnoses, emergency department visits, medications, laboratory tests) (32). By turning routine encounters into a population-wide dataset, this empirical data enables evidence-based decision-making and policy planning.

This systemic capacity for data generation should be matched with an innovative funding and delivery model for community health programmes. For instance, a microgrant programme in rural Australia uses health-service-funded grants to empower community members to lead place-based health promotion activities, such as social connection projects (33). This model of earmarked funding can be further strengthened by a tiered workforce approach that optimises operational reach and manages manpower expense. A study in Japan demonstrates this, where nurses, dietitians and researchers co-designed a dietary programme that was subsequently delivered by trained community health worker volunteers, successfully improving dietary variety in older adults (34). This combined approach directly breaks the cycle of ‘high manpower costs’ and ‘low perceived profitability’ by ensuring providers have the stable, upfront resources needed to deliver a safety-critical service, transforming it from a financially precarious project into an institutionalised public health function.

Ultimately, the experiences of Singapore and other systems converge on a single principle: effective scaling requires moving beyond funding discrete programmes to investing in the foundational pillars of the prevention ecosystem. Addressing these interconnected gaps is the necessary step to make falls prevention a scalable and sustained reality.

### 4.1 Limitations and future research

This study has several limitations. The primary limitation is that the findings are based solely on the perspectives of providers and system-level stakeholders; older adults themselves were not engaged in this FPI workshop. This was a deliberate choice to prevent potential social desirability bias, where older adults might hesitate to express negative views or preferences in the presence of clinicians and policymakers who represent the service provision system. On top of that, while the stakeholders represented key community providers, the sample size of 14 individuals from seven organisations, though diverse, may not capture the full spectrum of operational realities across all community care providers in Singapore. Finally, the CRT and the identified root causes, while rigorously constructed from stakeholder input, represent a hypothesised model of the system that requires further validation. Other limitations include that this was done in the context of Singapore, however the issues are likely to extend to other ageing countries with similar healthcare infrastructure.

This root cause analysis represents the foundational first step to a broader FPI approach. By identifying the core systemic barriers, this work establishes the diagnostic basis for subsequent phases, which can cluster community providers by their specific challenge profiles and co-develop tailored implementation toolkits to match their unique operational contexts. Future research must empirically test the proposed CRT and validate it with a broader group, including frontline staff and vendors. Following this, more rounds of FPI workshops should be held to develop an implementation toolkit based on the validated model. The subsequent step is to co-design and pilot these toolkit interventions, measuring their impact on root causes and FPPs sustainability.

## 5 Conclusion

In conclusion, this study identified the fundamental systemic barriers hindering the scale-up of evidence-based FPPs within Singapore’s community care landscape. Through a root cause analysis framework, we identified five root causes: (1) no systematic effort to inform older adults about falls and falls prevention, (2) lack of training resources, (3) insufficient funding, (4) a lack of both formal and informal data on demand for FPPs, and (5) high manpower costs to conduct FPPs. These findings manifest as three fundamental systemic gaps: an information gap that obscures need, a resource or capacity gap that cripples delivery, and an economic gap that renders programmes unsustainable for providers. Such gaps are not unique to Singapore but reflect a broader implementation challenge commonly observed in preventive health interventions, highlighting the importance of supportive ecosystems to enable scale.

## Data Availability

The raw data supporting the conclusions of this article will be made available by the authors, without undue reservation.

## References

[1] American Geriatrics Society. Guideline for the prevention of falls in older persons. J Am Geriatr Soc. (2001) 49:664–72. doi: 10.1046/j.1532-5415.2001.49115.x, 11380764

[2] O’Loughlin JL Robitaille Y Boivin JF Suissa S. Incidence of and risk factors for falls and injurious falls among the community-dwelling elderly. Am J Epidemiol. (1993) 137:342–54. 10.1093/oxfordjournals.aje.a116681 8452142

[3] Lee D Tak SH. A concept analysis of fear of falling in older adults: insights from qualitative research studies. BMC Geriatr. (2023) 23:651. doi: 10.1186/s12877-023-04364-5, 37821830 PMC10568775

[4] Dautzenberg L Beglinger S Tsokani S Zevgiti S Raijmann RCMA Rodondi N . Interventions for preventing falls and fall-related fractures in community-dwelling older adults: a systematic review and network meta-analysis. J Am Geriatr Soc. (2021) 69:2973–84. doi: 10.1111/jgs.17375, 34318929 PMC8518387

[5] Montero-Odasso M van der Velde N Martin FC Petrovic M Tan MP Ryg J . World guidelines for falls prevention and management for older adults: a global initiative. Age Ageing. (2022) 51:afac205. doi: 10.1093/ageing/afac205, 36178003 PMC9523684

[6] Xu Q Ou X Li J. The risk of falls among the aging population: a systematic review and meta-analysis. Front Public Health. (2022) 10:902599. doi: 10.3389/fpubh.2022.902599, 36324472 PMC9618649

[7] Saunders S D’Amore C Hao Q Richardson J. Risk factors for falls in community-dwelling older adults: an umbrella review. J Am Med Dir Assoc. (2025) 26:105765–5. doi: 10.1016/j.jamda.2025.105765 40651502

[8] Boright LE Arena SK Wilson CM McCloy L. The effect of individualized fall prevention programs on community-dwelling older adults: a scoping review. Cureus. (2022) 14:e23713. doi: 10.7759/cureus.23713, 35510013 PMC9060767

[9] Cameron ID Dyer SM Panagoda CE Murray GR Hill KD Cumming RG . Interventions for preventing falls in older people in care facilities and hospitals. Cochrane Database Syst Rev. (2018) 2020:CD005465. doi: 10.1002/14651858.cd005465.pub4, 30191554 PMC6148705

[10] Clemson L Stark S Pighills AC Fairhall NJ Lamb SE Ali J . Environmental interventions for preventing falls in older people living in the community. Cochrane Database Syst Rev. (2023) 2023:CD013258. doi: 10.1002/14651858.CD013258.pub2, 36893804 PMC9998238

[11] Hopewell S Copsey B Nicolson P Adedire B Boniface G Lamb S. Multifactorial interventions for preventing falls in older people living in the community: a systematic review and meta-analysis of 41 trials and almost 20 000 participants. Br J Sports Med. (2020) 54:1340–50. doi: 10.1136/bjsports-2019-100732, 31434659 PMC7606575

[12] Sherrington C Fairhall NJ Wallbank GK Tiedemann A Michaleff ZA Howard K . Exercise for preventing falls in older people living in the community. Cochrane Database Syst Rev. (2020) 2019:CD012424. doi: 10.1002/14651858.CD012424.pub2, 30703272 PMC6360922

[13] Albornos-Muñoz L Moreno-Casbas MT Sánchez-Pablo C Bays-Moneo A Fernández-Domínguez JC Rich-Ruiz M . Efficacy of the Otago exercise Programme to reduce falls in community-dwelling adults aged 65-80 years old when delivered as group or individual training. J Adv Nurs. (2018) 74:1700–11. doi: 10.1111/jan.13583, 29633328

[14] Nilsen P Bernhardsson S. Context matters in implementation science: a scoping review of determinant frameworks that describe contextual determinants for implementation outcomes. BMC Health Serv Res. (2019) 19:189. doi: 10.1186/s12913-019-4015-3, 30909897 PMC6432749

[15] Tsutsui T Muramatsu N. Japan’s universal long-term care system reform of 2005: containing costs and realizing a vision. J Am Geriatr Soc. (2005) 55:1458–63. doi: 10.1111/j.1532-5415.2007.01281.x, 17767690

[16] Brach JS Juarez G Perera S Cameron K Vincenzo JL Tripken J. Dissemination and implementation of evidence-based falls prevention programs: reach and effectiveness. J Gerontol A Biol Sci Med Sci. (2021) 77:164–71. doi: 10.1093/gerona/glab197, 34244780 PMC8751789

[17] Frieden TR. Six components necessary for effective public health program implementation. Am J Public Health. (2014) 104:17–22. doi: 10.2105/AJPH.2013.301608, 24228653 PMC3910052

[18] Dopp AR Narcisse MR Mundey P Silovsky JF Smith AB Mandell D . A scoping review of strategies for financing the implementation of evidence-based practices in behavioral health systems: state of the literature and future directions. Implementation Res Prac. (2020) 1:263348952093998. doi: 10.1177/2633489520939980, 37089129 PMC9924261

[19] Endalamaw A Khatri RB Erku D Zewdie A Wolka E Nigatu F . Barriers and strategies for primary health care workforce development: synthesis of evidence. BMC primary care. (2024) 25:99. doi: 10.1186/s12875-024-02336-1, 38539068 PMC10967164

[20] Yeo C Ying Y Lee Y Lim S Quek CY. A review of elderly injuries seen in a Singapore emergency department. Singapore Med J. (2009) 50:278. 19352571

[21] Ministry of Health Supporting health and well-being across every life stage (2025). Available online at: https://www.moh.gov.sg/newsroom/supporting-health-and-well-being-across-every-life-stage/ (Accessed March 3, 2026).

[22] Age Well SG. Active Ageing. Singapore: Age Well SG. (2026).

[23] Matchar DB Patwardhan M Samsa GP Haley WE. Facilitated process improvement: an approach to the seamless linkage between evidence and practice in CKD. Am J Kidney Dis. (2006) 47:528–38. doi: 10.1053/j.ajkd.2005.11.016, 16490633

[24] Goldratt EM. Theory of Constraints. Croton-on-Hudson (NY): North River Press (1992).

[25] Dettmer HW. The logical Thinking process: a Systems Approach to complex problem Solving. Boca Raton (FL): CRC Press (2007).

[26] Dettmer HW. Goldratt's Theory of Constraints: a Systems Approach to Continuous Improvement. Milwaukee (WI): ASQC Quality Press (1997).

[27] Goldratt EM Cox J. The goal: a process of Ongoing Improvement. 2nd ed. Croton-on-Hudson (NY): North River Press (1984).

[28] Reardon CM Damschroder LJ Ashcraft LE Kerins C Bachrach RL Nevedal AL . The consolidated framework for implementation research (CFIR) user guide: a five-step guide for conducting implementation research using the framework. Implement Sci. (2025) 20:39–9. doi: 10.1186/s13012-025-01450-7, 40819067 PMC12357348

[29] Damschroder LJ Aron DC Keith RE Kirsh SR Alexander JA Lowery JC. Fostering implementation of health services research findings into practice: a consolidated framework for advancing implementation science. Implement Sci. (2009) 4:50. doi: 10.1186/1748-5908-4-50, 19664226 PMC2736161

[30] Ye P Jin Y Er Y Yin X Yao Y Li B . Perceptions of facilitators and barriers to implementation of falls prevention programs in primary health care settings in China. JAMA Netw Open. (2022) 5:e2228960. doi: 10.1001/jamanetworkopen.2022.28960, 36018587 PMC9419020

[31] Pantong U Trapero I Jareaprapal U. Analysis and prevention of falls among community-dwelling older adults in southern Thailand. J Adv Nurs. (2024) 80:2121–36. doi: 10.1111/jan.15945, 37950381

[32] Yang A Shi M Lai ESH Yu J Luk AOY Ma RCW . A novel electronic-health-record based, machine-learning model to predict 1-year risk of fall hospitalisation in older adults: a Hong Kong territory-wide cohort and modelling study. Age Ageing. (2025) 54:afaf285. doi: 10.1093/ageing/afaf285 PMC12510402 41066674

[33] Conlin M McLaren D Spelten E MacDermott S. Community-driven health promotion: evaluation of a rural microgrant program. Health Expect. (2024) 27:e70098. doi: 10.1111/hex.70098, 39523704 PMC11551473

[34] Murayama H Taguchi A Spencer MS Yamaguchi T. Efficacy of a community health worker–based intervention in improving dietary habits among community-dwelling older people: a controlled, crossover trial in Japan. Health Educ Behav. (2020) 47:47–56. doi: 10.1177/1090198119891975, 31933395

