# Supplementary material for: Implementation barriers in Singapore’s community-based falls prevention programmes: a root cause analysis
Source: Front Public Health. 2026 Oct 12;14:1913505. doi: 10.3389/fpubh.2026.1913505 (PMC13696527; doi:10.3389/fpubh.2026.1913505)
Supplement: Supplementary file 1 [file Table_1.docx]

# Supplementary Materials

***Supplementary Table S1: Overview of Community Falls Prevention and Healthy Ageing Programmes in Singapore***

| **Programme Name** | **Lead Organisation and Programme Location** | **Target Population** | **Duration & Frequency** | **Key Components** | **Outcome** |
| --- | --- | --- | --- | --- | --- |
| Combat Age-Related Loss of Muscle (CALM) [1, 2] | Active Health, Sport  Singapore, Singapore Ministry of Culture, Community and Youth; Conducted island-wide across nine Active Health Hubs | CALM 1.0: Older adults aged 60 and above | CALM 1.0: 8-weekly 60-minute sessions | CALM 1.0: Physical assessments, resistance exercises, domain sharing | Muscle building |
|  |  | CALM 2.0: Older adults aged 60 and above who completed CALM 1.0 | CALM 2.0: 6-weekly 60-minute sessions | CALM 2.0: Strength training principles, gym machine orientation |  |
| Healthy Ageing Promotion Programme For You (HAPPY) [3] | Singapore Health Promotion Board; Conducted island-wide with local community partners | Older adults aged 60 and above who can walk independently or with walking aid | 12 to 24 weekly or biweekly 60-minute sessions (programme frequency varies across sites) | Multicomponent exercises incorporating physical, cognitive and social activities conducted by trained volunteers | Improvement in cognition |
| SHARE Falls Prevention Programme [4] | Sengkang General Hospital; Conducted within the North-East precincts of Singapore with selected AAC partners | Older adults in the community | 4 sessions | Educational sessions by allied health professionals including occupational therapy (home safety), physiotherapy (exercise), podiatry (footcare), and dietetics (nutrition) | Improving falls prevention behaviours |
| Steady Lah! Programme [5] | Singapore Health Promotion Board; Conducted island-wide with local community partners | Older adults aged 50 and above who can walk independently | 12-weekly 120-min sessions | Low-to-moderate intensity progressive strength and balance exercises with interactive lectures on diet and nutrition conducted by layperson (e.g. fitness trainers and workshop facilitators) | Improving overall physical health |
| Steps to Avoid Falls in the Elderly - a TECHnology Enhanced Intervention (SAFE-TECH) [6] | Centre for Ageing Research & Education (CARE), Duke-NUS Medical School; Conducted island-wide at thirteen sites with selected Active Ageing Centre (AAC) partners | Community-dwelling older adults aged 60 and above at high risk of falls | 24-biweekly 120-minute sessions | Tailored multi-domain exercises including strength balance, flexibility, coordination and endurance, with weekly educational sessions on risk factor management and personalised feedback, all conducted by physiotherapists and aides | Falls prevention |

***Supplementary Table S2: The Categories of Legitimate Reservation (CLR) [7]***

| **CLR Category** | **Probing Question** |
| --- | --- |
| 1. Clarity | Are all terms and entities clearly defined? |
| 2. Entity Existence | Do the proposed entities actually exist in the described context? |
| 3. Causality Existence | Is there a logical mechanism supporting the cause-effect link? |
| 4. Cause Insufficiency | Is the cause sufficient to produce the effect? |
| 5. Additional Cause | Could other causes be responsible? |
| 6. Cause-Effect Reversal | Could the supposed effect actually be the cause? |
| 7. Predicted Effect Existence | Are all inherent side effects acknowledged? |
| 8. Tautology | Is any circular logic present? |

***Supplementary Table S3: Illustrative Examples of CLR Validation During the FPI Workshop***

| **Causal Linkage Proposed by Stakeholders** | **CLR Reservation Applied** | **Probing Question** | **Clarification / Revision** |
| --- | --- | --- | --- |
| "Older adults do not attend falls prevention programmes (FPP) because they are unaware of available programmes." | 3. Causality Existence | Is there a logical mechanism supporting this cause–effect link? | Stakeholders clarified that awareness alone is insufficient; even when informed, older adults may not attend due to perceived irrelevance or cost concerns.  The causal link was refined to: "Older adults do not attend FPPs due to a combination of lack of awareness of availability and benefits, perceived low value of FPP, perceived low personal fall risk, and cost concerns." |
| "AACs lack sustained FPPs because they have insufficient funding." | 4. Cause Insufficiency | Is this cause sufficient to produce the effect? | Stakeholders noted that funding alone does not explain the lack of sustained programmes; even when funding is available, AACs lack trained staff to deliver FPPs and are not held accountable for FPP-related KPIs.  The causal link was revised to: "AACs lack sustained FPPs due to insufficient manpower, lack of staff knowledge and training, and omission of FPPs as a KPI by funders." |

***Supplementary Figure S1: Facilitated Process Improvement (FPI) Workshop Activity 1 Worksheet***

***Supplementary Figure S2: Full CRT illustrating the factors influencing the delivery and sustainability of falls prevention programmes, from the perspective of Integrated Community Care Partners (ICCPs) in Singapore.***

## References

1. Combat Age-Related Loss of Muscle (CALM) 1.0 [Internet]. Activesgcircle.gov.sg. 2026. Available from: https://www.activesgcircle.gov.sg/activehealth/our-programmes/combat-age-related-loss-of-muscle-calm-10
2. Combat Age-Related Loss of Muscle (CALM) 2.0 [Internet]. Activesgcircle.gov.sg. 2026. Available from: https://www.activesgcircle.gov.sg/activehealth/our-programmes/combat-age-related-loss-of-muscle-calm-20
3. Merchant RA, Tsoi CT, Tan WM, Lau W, Sandrasageran S, Arai H. Community-Based Peer-Led Intervention for Healthy Ageing and Evaluation of the ‘HAPPY’ Program. The journal of nutrition, health & aging. 2021, 25(4):1. https://doi.org/10.1007/s12603-021-1606-6
4. SHARE [Internet]. Sengkang General Hospital. 2026 Available from: https://www.skh.com.sg/community-care/community-health-services/skh-allied-health-population-health/share
5. Jayaprakash KP, Ngo R, Lee E, Chng PL, Lee H, Chua S, et al. Case study: lessons learned from a community-based early frailty intervention programme in Singapore. Frontiers in public health. 2024, 12: p. 1445347. https://doi.org/10.3389/fpubh.2024.1445347
6. Lai WX, Koh V, Goh JW, Tan KY, Tan KZ, Pai SGS, Taylor WR, Visaria A, Singh NB, Chan AWM, Matchar DB. Steps to Avoid Falls in the Elderly - a TECHnology Enhanced Intervention (SAFE-TECH) study: randomized controlled trial protocol for a community-based, multi-component fall prevention program. BMC Public Health. 2024 Dec 18;24(1):3464. doi: 10.1186/s12889-024-20947-2.
7. Dettmer HW. Systems Thinking - And Other Dangerous Habits. Virtualbookworm.com Publishing; 2021.
8. Simpson JM, Darwin C, Marsh N. What are older people prepared to do to avoid falling? A qualitative study in London. British Journal of Community Nursing. 2003 Apr;8(4):152–9. 10.12968/bjcn.2003.8.4.11190
9. Fernandes J, Fernandes S, Almeida A, Vareta D, Miller C. Older Adults’ Perceived Barriers to Participation in a Falls Prevention Strategy. Journal of Personalized Medicine. 2021 May 23;11(6):450. 10.3390/jpm11060450
10. Currie DW, Thoreson SR, Clark L, Goss CW, Marosits MJ, DiGuiseppi C. Factors Associated With Older Adults’ Enrollment in Balance Classes to Prevent Falls: Case-Control Study. 2020 Aug 1;39(8):908–14. 10.1177/0733464818813022
11. Kramer BJ, Vivrette RL, Rubenstein LZ. Engaging Community-Based Organizations in Fall Prevention Education. Gerontology & Geriatrics Education. 2011 Apr;32(2):182–96. https://doi.org/10.1080/02701960.2011.572209.
12. Vlaeyen E, Stas J, Leysens G, Van der Elst E, Janssens E, Dejaeger E, et al. Implementation of fall prevention in residential care facilities: A systematic review of barriers and facilitators. International Journal of Nursing Studies. 2017 May 1;70:110–21.10.1016/j.ijnurstu.2017.02.002
13. Fernández MG, Pantiga MM, Fernández CP, Muñoz LA, Guerrero CG. Fall assessment and intervention among community-dwelling older people in a primary health care center in Spain: a best practice implementation project. JBI Evidence Implementation. 2024 Jun 26. 10.1097/XEB.0000000000000440
14. Wang H, Jin Y, Wang D, Zhao S, Sang X, Yuan B. Job satisfaction, burnout, and turnover intention among primary care providers in rural China: results from structural equation modeling. BMC Family Practice. 2020 Jan 15;21(1). https://doi.org/10.1186/s12875-020-1083-8
15. Yang G, Wei H, Wan L, Dong H, Liang X, He Y. Curvilinear relationship between burnout and work engagement among staff in community services for the elderly: A correlation study. Frontiers in Public Health. 2022 Jul 22;10. 10.3389/fpubh.2022.939649
16. Kwon J, Lee Y, Young T, Squires H, Harris J. Qualitative research to inform economic modelling: a case study in older people’s views on implementing the NICE falls prevention guideline. BMC Health Services Research. 2021 Sep 28;21(1). https://doi.org/10.1186/s12913-021-07056-1
